# The Role of Coffee Silver Skin against Oxidative Phenomena in Newly Formulated Chicken Meat Burgers after Cooking

**DOI:** 10.3390/foods10081833

**Published:** 2021-08-08

**Authors:** Maria Martuscelli, Luigi Esposito, Dino Mastrocola

**Affiliations:** Faculty of Bioscience and Technology for Food, Agriculture and Environment, University of Teramo, Via R. Balzarini 1, 64100 Teramo, Italy; lesposito2@unite.it (L.E.); dmastrocola@unite.it (D.M.)

**Keywords:** coffee by-products, chicken burger, meat formulation, cooking yield, volatile compounds, warmed off-flavours

## Abstract

Coffee Silver Skin (CSS) is the unique by-product discarded after the roasting of coffee beans. This research aimed to evaluate the effect of two levels of CSS (1.5% and 3%) added as a natural ingredient in new formulations of chicken meat burgers. This is one of the first studies proposing a “formulation approach” to control the emergence of off flavours after meat cooking. Physical, chemical, and sensory analyses were carried out, within the CSS content and the evolution of volatile organic compounds in different samples. Newly formulated chicken burgers could limit food waste, while also becoming a source of fibres, minerals, and bioactive molecules. CSS limited weight losses (after cooking process) to 10.50% (1.5% addition) and 11.05% (3% addition), significantly lower (*p* < 0.01) than the control (23.85%). In cooked burgers, the occurrence of hexanal was reduced from 55.1% (CTRL T_0_) to 11.7% (CSS T_0_ 1.5%) to 0 (CSS T_0_ 3%). As for the limitation of off-flavours, CSS also showed good activity, contrasting with the emergence of octanal, alcohols and other markers of lipid oxidation. From the sensory test carried out, the volatile profile of CSS does not seem to impair the flavour of burgers, though at higher percentages hydrocarbons and pyrazines are traceable. The thiobarbituric acid reactive substances (TBARS assay confirmed the protective effect of CSS against oxidation.

## 1. Introduction

Minimally processed raw by-products are available in large quantities and their re-utilization might be enhanced to recover bioactive compounds, on top of their promising technological properties [1]. Many valuable molecules such as phenolic acids, carotenoids, and flavonoids can mitigate oxidation occurrence, so there is an increased demand for new methods and technologies to recover and use these [2]. Many publications attest the positive role of by-products’ addition in meat formulations to limit oxidation occurrence [3,4].

Oswell et al. [5] explain how some unprocessed food components can help reducing the list of ingredients of a formulation, supporting trends towards the clean and green label. In fact, many by-products have technological properties, acting as additives and ingredients [6].

Coffee Silver Skin (CSS) is a thin layer tightly adherent to coffee seeds, present in all coffee species and impossible to separate when seeds are unroasted [7]. Among all the by-products of the coffee industry, CSS is unique in being discarded immediately after the roasting step [8]. 

Common features of CSS are high content in fibres (both soluble and insoluble), in minerals such as Calcium and Potassium, and in capability as an adsorbing material [2]. 

To our knowledge, there are no studies of the inclusion of untreated coffee silver skin (CSS) in meat-based foods such as chicken products. It is well known that poultry meat is easily oxidised; its content of monounsaturated fatty acids (MUFAs) and polyunsaturated fatty acids (PUFAs) (including phospholipids that are distributed in muscles and cellular membranes) make it the elective substrate for lipid oxidation [3]. Feeding of animals is responsible for many qualitative characteristics of meat, progressing to the eventual development of off-flavours (after slaughtering) [9,10]. In chicken products, clove, oregano, thyme, and sage were successfully used as natural substitutes of synthetic antioxidants [11]. Kim et al. [12] used residues of coffee brewing (spent coffee) as antioxidants against meat oxidation in raw and cooked samples. Recently Delgado-Ospina et al. [13] added cocoa pod husk flour, discovering an interesting application for quality improvement of frankfurters. Cooked and refrigerated meat products develop undesired rancidity and a great variety of off flavours known as warmed over flavours (WOF). These defects can also come about by heating ready to eat foods or high-processed meat-based items and can be present in many products where food remains exposed to light, oxygen, and heat for a long time (canteens, fast food outlets, collective restaurants). In the study by Lungu et al. [14], most respondents affirmed exposure to WOF defective foods, especially ready to eat meats; moreover, besides the reduced sensory quality, respondents confirmed a preference for defective foods due to their lower cost. The development of WOFs does not impair food safety, but a high oxidation rate affects the nutritional profile; therefore a huge quantity of safe food is discarded daily due to detrimental sensory defects. 

In this scenario, the present research investigated the chemical and physical properties of CSS against oxidative phenomena after the cooking of chicken burgers. This study points to the nutritional advantages of including CSS as a new ingredient for chicken burger recipes, while testing some technological functionalities. Lastly, we were able to evaluate the role of CSS on the shelf life of refrigerated cooked burger, focusing on the spreading of WOF and oxidation markers along with an analysis of the volatile compounds and a sensory test with trained panellists.

## 2. Materials and Methods

### 2.1. Coffee Silver Skin

Coffee silver skin (CSS) was received from the toasting plant Marcafè Torrefazione Adriatica s.p.a. (Giulianova, Italy). After 10 cycles of roasting (10 × 240 kg of roasted coffee), 3.3 kg of CSS were recovered. CSS used for this experiment was a blend of 5 arabica varieties (*Coffea arabica*) (India Arabica, India Cherry, Vietnam, India Mysore, and India Caracolito) and 5 robusta varieties (*Coffea canephora*) (India Parchment, Santos, Uganda CRV 18, Uganda CRV 17, Togo). CSS was ground at 10,200 rpm for 1 min (Bimby^®^, mod. TM 31, Wuppertal, Germany) until arriving at a particle size of 125–250 µm. Then, physical, and chemical analyses were carried out. CSS was kept frozen at −20 °C until analysis. Qualitative analyses were carried out and results were reported in our previous study [2].

### 2.2. Preparation of Burgers

Chicken breast fillets were purchased on the market (antibiotic-free, genetically modified organism-free diet, and high welfare/partial free range system meat).

Chicken burgers were obtained from 1 kg of fresh breast fillets with the addition of 1.4% salt and 5.0% water. These ingredients were cut and mixed for 2 min at 1800 rpm with Bimby^®^ mixer (Wuppertal, Germany), mod. TM 31, to obtain a perfectly homogenised blend.

From the whole mixture (meat, water, and salt) 3 batches were obtained: control, without any addition of coffee silver skin (CTRL), coffee silver skin addition of +1.5% (CSS 1.5%) and coffee silver skin addition of +3.0% (CSS 3.0%). CSS was added by mixing for 30 s at 500 rpm.

**Figure 1:**
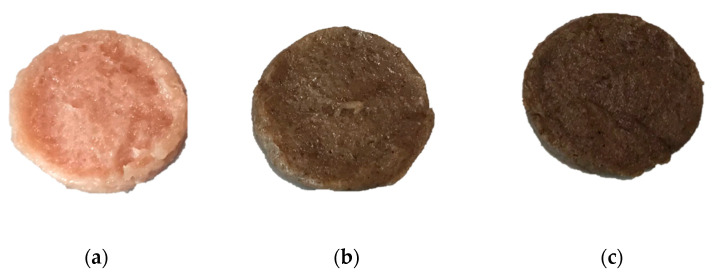
Uncooked burgers of the three experimental batches: (**a**) control (CTRL) without CSS additions; (**b**) with addition of +1.5% of coffee silver skin (CSS 1.5%); (**c**) with +3.0% of coffee silver skin (CSS 3.0%).

8 burgers (45 g each approximately) were prepared for each experimental batch (Figure 1).

**Figure 2:**
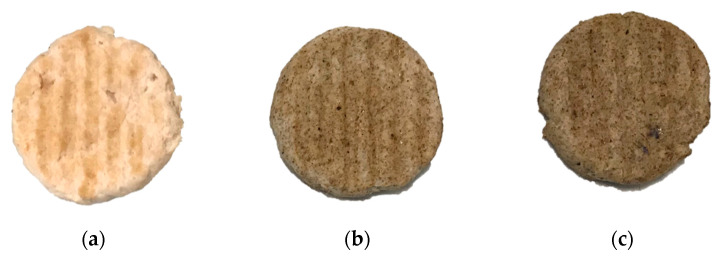
Cooked burgers of the three experimental batches: (**a**) control (CTRL); (**b**) with addition of +1.5% of coffee silver skin (CSS 1.5%); (**c**) with +3.0% of coffee silver skin (CSS 3.0%).

Then, burgers were cooked on an electrical griddle Bosch (München, Germany), mod. TFB4431V, potency 2000 W for 4 min until reaching an internal temperature of 90–92 °C. Cooked burgers are shown in Figure 2. After cooking, some burgers were eaten during the panel test, and others were left singularly covered with a plastic oxygen permeable film at +4 °C for 72 and 120 h. The utilization of this covering film was chosen to allow the permeability of O_2_ and thus the spreading of WOFs and other products of oxidation. Moreover, this condition is the closest to what can happen to consumers at home. The trial was replicated on another two independent occasions.

### 2.3. Physico-Chemical, Colour, and Compositional Analyses

The values of water activity (a_w_) were obtained with the Aqualab 4 TE kit (Court Pullman, WA, USA). Values of pH were taken with a pH meter (model 3510, Jenway, Stone, UK). All values were measured in triplicate.

Colour was determined in different locations of burger samples by a colorimeter CR-5 (Spectrally based, Konica Minolta, Tokyo, Japan) with D_65_ light source and observer 10°. Colour was expressed as L* (lightness, intensity of white colour), a* (+a, red; −a, green) and b* (+b, yellow; −b, blue) values. Samples were measured in triplicate and at least fifteen measurements were obtained for each batch. To better define the final color observed, the saturation index (chroma, C*) was calculated according to Formula (1).

C* = (a*^2^ + b*^2^)^1/2^ (1)

Proximate analysis on moisture, proteins, and ashes was obtained following the Association of Official Analytical Chemists procedure [15]. Total lipids were measured using a modification of the chloroform to methanol procedure described by Folch et al. [16].

The determination of such micronutrients as calcium, potassium, and total dietary fibers (TDF) was performed by calculating their presence in a 45 g burger at different formulations; the estimation of Ca, K, and TDF accounts of the values found in our previous work on the characterization of CSS [2]. Quantities found refer to the presence of a defined element and not on bioavailability. We used easy proportions to find percentages. 

Here, as example, we propose the calculation used for the calcium determination (2), (3) in 1.5% CSS formulation:

(2) 546.5 mg:100 g=3.3 g:x 

(3) $$x=\frac{\left(546.5\;\mathrm{mg}*100\;g\right)}{3.3\;g}=16.54\;\mathrm{mg}$$

where 546.5 mg is the Ca content in 100 g of CSS, 3.3 g is the amount of CSS in a 45 g burger, and 16.54 mg is the intake of Ca in a 45 g burger formulated with 1.5% of CSS.

### 2.4. Cooking Yield

The cooking yield parameter is a useful and practical tool to easily calculate the quantity of meat available for consumption after the cooking process. Uncooked samples were prepared and weighted singularly, then underwent the established cooking process and weighed again. This formula was used to arrive at the result (4)

(4) $$100-\left(\frac{\left(\mathrm{raw}\;\mathrm{burger}\;\mathrm{weight}-\mathrm{cooked}\;\mathrm{burger}\;\mathrm{weight}\right)}{\left(\mathrm{raw}\;\mathrm{burger}\;\mathrm{weight}\right)}\times 100\right)$$

### 2.5. Thiobarbituric Acid Reacting Substances (TBARS) Assay

A thiobarbituric reactant species test was carried out following the methods of Soyer et al. [17] with some modifications. Raw meat (25 g) was ground in 125 mL of pure water for 2 min to homogenise the mixture. From this, 5 mL were filtered and transferred in falcon tubes (15 mL) with 3 mL of a solution containing trichloroacetic acid (15%, *w*/*v*) and thiobarbituric acid (80 mM) in HCl 0.25 N. Samples underwent a centrifugation step (2000 rpm for 5 min) to precipitate proteins. After centrifugation, 3 mL were transferred in tapped glass tubes and kept at 40 °C for 90 min.

Samples obtained were read at 532 nm with a spectrophotometer UV-VIS (Jenway, Stone, UK) after a further filtration with filters 0.45 µm. All samples were read in double, and data were expressed as mean ± standard deviation.

The calibration curve was obtained by using a 1,1,3,3-tetraetoxypropane (Sigma-Aldrich, St. Louis, MO, USA, ≥96%) in methanol, at a concentration range of 0.625–20 µM.

### 2.6. Volatile Compounds (VOCs)

The experimental plan was designed to have triplicate samples of each formulation of T_0_ cooked samples, T_72_ and T_120_ samples. Cooked samples T_0_ were immediately chopped and put in glassy vials of 20 mL capacity (Perkin Elmer, Waltham, MA, USA) with approximately 3 g of meat each, tightly closed and stocked at −40 °C, assuring the highest headspace, until gas chromatograph mass spectrometer (GC-MS) analysis. GC-MS analysis was performed with a gas chromatograph (Clarus 580, Perkin Elmer, Waltham, MA, USA) coupled with a mass spectrometer (SQ8S, Perkin Elmer Waltham, MA, USA). Other samples were left in refrigerated conditions for the time required to obtain T_72_ and T_120_, then carefully chopped and stocked in 20 mL vials at −40 °C, until GC-MS analysis.

The GC-MS analysis followed the method proposed by Qi et al. [18] with some modifications. Vials were left for 1 h at room temperature, then put in a water bath at 50 °C for 20 min. Volatiles from meat were extracted with a headspace solid phase microextraction fibre (SPME 65 μm Polydimethylsiloxane/Divinylbenzene (PDMS/DVB); Supelco, Bellofonte, PA, USA) and collected for 30 min at 40 °C, then inserted into the GC injector and desorbed for 3 min at 250 °C. Volatile compounds were separated on a Capillary GC column ZB- Semi Volatiles (30 m length, 0.25 mm internal diameter, 0.25 μm film thickness: Phenomenex, Torrance, CA, USA). The oven temperature was maintained for 3 min at 40 °C, increased at 3 °C/min to 70 °C, then at 5 °C/min to 180 °C, then at 10 °C/min to 260 °C, and maintained for 5 min at 260 °C. Helium was the carrier gas with a constant flow of 1 mL/min. The mass-selective detector was operated in the electron impact mode (70 eV) and full scan mode (35–500 *m*/*z* range). The identification was performed using the National Institute of Standards and Technology mass spectral library (NIST Mass Spectral library, Search Program version 2.0, National Institute of Standards and Technology, U.S. Department of Commerce, Gaithersburg, MD, USA).

### 2.7. Descriptive Sensory Analysis of WOF Assessment, Rancidity and Extraneous Flavours

Chicken burger samples were evaluated for four classes of descriptors grouped as: odour, flavour, taste, and aftertaste [19]. The vocabulary used for descriptors comes from the review of recent literature about rancidity and warmed off-flavours (WOF) assessment in meat products [20,21,22,23,24].

A panel group of six women and two men from twenty to fifty years old was trained for evaluation of quality assessment of meat burgers. After their recruitment, panellists were screened for their ability to distinguish odours and tastes, then were trained for vocabulary development through a series of triangular tests (ISO), 8586:2012 [25]. Training duration was 80 h, including familiarization with relevant descriptive terms and ways of perceiving the selection and quantification of the sensory characteristics of cooked meat, as well as the use of intensity scales (ISO) 4121:2003 [26].

Panellists were asked to taste cooked burgers (T_0_) and cooked burgers refrigerated at +4 °C for 72 h. Samples of 120 h at +4 °C were not tasted to avoid any microbial contamination. A hot bath at 72 °C was used to heat up to the core temperature of 70 °C. Panellists were provided with individual templates, where descriptors were grouped per section. They used a scale from 1 to 5 where 1 meant the absence of the attribute while 5 the maximum rate. Pie shaped pieces of warm burgers were quickly served to panellists trying to maintain the temperature between 70 and 60 °C as recommended by [27]. Samples were codified with random numbers to avoid external influences on liking rating of panellists. Meat pieces were served on white plates. All sensory tests and training sessions were carried out in the sensory laboratory of the University of Teramo that fulfils the required standards for these analyses according to (ISO) 8589:2007 [28].

### 2.8. Statistical Analysis

All determinations were done in triplicate. Means and relative standard deviations were calculated. Analysis of variance (ANOVA) was performed to test the significance of the effects of the factor variables (formulation, time of storage); differences among means were separated by the least significant differences (LSD) test. 

Statistical analysis of data was performed using XLSTAT software version 2019.1 for Microsoft Excel (Addinsoft, New York, NY, USA). All results were considered statistically significant at *p* < 0.05.

## 3. Results and Discussion

Results of qualitative characteristics (proximate composition, colour, pH, a_w_) of uncooked and immediately cooked burgers are shown and discussed in the first section, highlighting significant differences among treatments. Data shown and discussed in the second section (TBARS, VOCs, sensory analysis) evidence the significant differences between sampling times with respect to the oxidative phenomena occurred in the refrigerated cooked chicken burger samples.

### 3.1. Effect of CSS on the Qualitative Characteristics of Burger Samples

#### 3.1.1. Compositive Characteristics

Results from proximate compositional analyses of meat are shown in Table 1 (uncooked burgers); data agree with the literature [29,30]. Generally, macro-elements checked do not change so much. Moisture, proteins, and lipids remained at around their normal values; ashes had a small significant increase reaching the highest point of 2.69% for 3% CSS sample. TDF for samples (calculated as reported in Section 2.3) are 1.70% and 3.40%, for 1.5% and 3% CSS, respectively.

pH of breast fillets used for the burger production was 5.93 ± 0.04, not differing with reported values which indicate values around 5.89–6.00 [31]. Formulated burgers (uncooked and cooked) registered pH values, shown in Table 2, in line with other sources [32]. Cooked burgers had similar pH and a_w_ values. 

In eating a newly formulated burger (45 g) with a CSS inclusion of 1.5%, 16.54 mg of calcium and 65.7 mg of potassium can be assumed. Harvard Health Publishing in 2019 reviewed the daily intake of calcium for women between 50 and 71 years old fixing this at 1200 mg [33]. The same value was established by the National Institute of Health (NIH) which also defines limits [34] for men at 1000 mg. Potassium was fixed at 3400 mg and 2600 mg for males and females from 19 to 50 years old, respectively.

For marketing within the European Union (EU), it is very important to define whether by-products, such as CSS, need to obtain an approval as ingredient for novel foods [34], with special reference to legal status within the EU and potential options for producers to obtain approval according to Novel Food [35,36,37].

#### 3.1.2. Cooking Yield

The cooking yield allows calculation of how much water and fats a food item loses after a cooking process. The US Department of Agriculture (USDA) in 2014 [38] released a table of cooking yield and retention factors for many meat products. These factors can be used to calculate nutritional values where analytical data for cooked foods are unavailable. Obviously, meat represents an important class of cooked foods and in this way the cooking yield covers an important aspect. Beside this, the cooking yield parameter tells us how much in terms of weight a formulation has lost, and this is also an index of profitability.

Table 2 shows that the addition of coffee by-product allowed an increase in cooking yield (%) in respect to the control. While the control lost 23.85% of its initial weight, CSS addition limited this loss to just 10.50% (+1.5% CSS addition) and 11.06% (+3% CSS addition). This trait of CSS comes from good water holding capacity (WHC) and oil holding capacity (OHC).For CSS, values of WHC 5.11 ± 0.20 and 5.5 ± 0.2 were found; for OHC, these were 4.72 ± 0.10 and 4.8 ± 0.2 [39,40,41,42,43]. The increased cooking yield also marked a significant difference (*p* < 0.01) between CTRL and CSS added burgers, while burgers with CSS, at both percentages, were similar as regards cooking yield.

Losses are limited even with little addition, registering a high capacity and a possible increase in economical revenue.

#### 3.1.3. Colour

Breast fillets used to formulate burgers had a value L* of 45.91, on average according to Ziober et al. [43], L* > 53 denoted pale soft exudative (PSE), L* < 44 is analogous to dark firm dry (DFD), and 44 ≤ L* ≤ 53 is normal meat. Colour values of burgers are in line with what Longato et al. [4] have found in their study on chicken burgers with the addition of hazelnut skin; their results on uncooked (control) samples are L* a* b* values of 53.83 ± 4.48, 0.30 ± 0.69, 9.12 ± 1.8, and 64.98 ± 2.55, 1.64 ± 0.55, 15.48 ± 1.15 for cooked burgers. Data on the colour determination (uncooked and cooked samples) are reported in Table 3.

CSS is easily reducible to a fine crumb and this determined a uniform distribution. Burgers with CSS are in fact darker, more yellow, and redder with respect to the control. Unfortunately, no studies are available to compare these data. Chroma (C*) values for uncooked and cooked chicken burgers were searched by de Oliveira et al. [44], who added chia seeds to a blend of breast and thigh chicken skinless meat and pork backfat. They reported levels of 14.0 ± 4.8 (raw samples), and 16.8 ± 2.4 (grilled samples). As in our case, burgers were darker than control. Conversely, their data do not show higher saturation. C* values of burgers here analysed are shown in Table 3. Generally, the addition of CSS increased the C* value meaning a higher saturation and, thus, a more vivid colour after cooking.

### 3.2. Effect of CSS on the Shelf Life of Cooked Chicken Burger Samples

#### 3.2.1. Thiobarbituric Acid Reacting Substances (TBARS) Test

**Figure 3:**
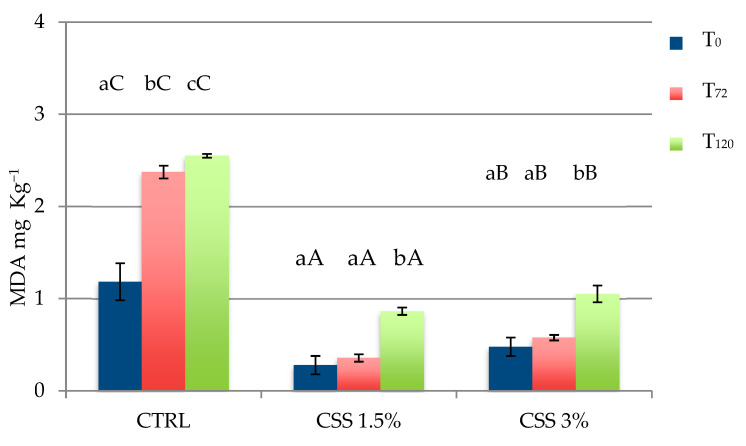
Results of Thiobarbituric acid reacting substances (TBARS) test, expressed as malondialdehyde (mg MDA kg^−1^) in control (CTRL) and burgers formulated with CSS (1.5% and 3%, respectively), immediately after cooking (T_0_) and after refrigerated storage (at 4 °C, for 72 and 120 h, T_72_ and T_120_ respectively). Results are expressed as means ± standard deviations. Different lowercase letters indicate significant differences (*p* < 0.05) among storage time of each batch; different uppercase letters indicate significant differences (*p* < 0.05) among different batches, at same storage time.

The TBARS values of cooked samples are presented in Figure 3. Generally, over time TBARS values increase in all the cases, but the CTRL set showed significantly higher values immediately after cooking (*p* < 0.05), as well as a statistically significant increase during the refrigerated storage (*p* < 0.05). In all burgers with CSS, TBARS mean values were lower than the acceptance limit of TBARS for rancidity (1.0 mg MDA(Malondialdehyde)/kg) [45] until 72 h; after 120 h CSS3% showed a TBARS mean value near to the critical content, while this limit was exceeded in all samples without CSS. 

The TBARS test is helpful for a first screening of the oxidation rate of a food, but it does not discriminate which kind of oxidation is occurring. We cannot be sure if what is observed with this assay comes from lipid oxidation itself, or if proteins too have taken part in the process and are the principal cause starting the reactions. As is known, chicken meat is poor in lipids that are mainly unsaturated fatty acids which are the elective substrate for the oxidation. Moreover, [46] have searched for the lipidic profile of CSS and their results show a small content of lipids, which are mainly saturated fatty acids (SFAs). So, one could refer all the defective odours and tastes to the lipidic oxidation complex of reactions. Unfortunately, the oxidation process is very unstable and sometimes unpredictable. Besides lipids, proteins and iron ions boost the process within other factors (rise in temperature, oxygen and light exposure, salt addition, etc.) making TBARS not such an affordable method to establish the lipidic oxidative status of a food [47,48].

Anyway, from our results we can imagine that the contribution of CSS to the global oxidation burgers and its lipids increment are almost zero, while its protective effect seems to be promising. This may depend on the high content in phenolic and bioactive species.

#### 3.2.2. Volatile Compounds (VOCs) and Warmed Off-Flavours (WOF) in Chicken Burgers

Among analyses used in this work, GC-MS analysis was used to trace markers of oxidation as a more reliable method than TBARS or any other faster, but less accurate, method. Results show a complex profile of compounds emerging from oxidative phenomena, Maillard reaction occurrence, and by-products addition. Table 4 contains all the volatile compounds found in CSS-containing samples. Data depicted refer to T_0_ and T_72_ samples. Chromatograms are shown as Supplementary Material (Figures S1–S3).

According to Chen et al. [49], most of the typical odorants from cooked chicken meat are caused by phospholipids oxidation/degradation that led to the formation of long-chain aldehydes such as hexanal, (Z)-2-decenal and (E)-2-decenal. In any case, even if responsible for WOF development, these aldehydes are key aroma compounds of freshly cooked chicken meat. CSS samples showed these classes even if ketones and esters were not found. T_0_ 1.5% added samples had hydrocarbons as the first class traced, followed by aldehydes, nitrogen containing compounds and alcohols. T_0_ 3% added samples showed aldehydes at first place followed by Nitrogen containing compounds, alcohols, hydrocarbons, and other compounds. T_72_ containing 1.5% of CSS showed nitrogen containing compounds, hydrocarbons, other compounds, aldehydes, alcohols, and ketones, while 3% addition showed aldehydes, hydrocarbons, alcohols, other compounds, and Nitrogen containing compounds.

In general, patties tested in this study, especially CTRL, seem to have a small compounds presence if compared with other articles [50,51,52]. To our knowledge, this is one of the first studies where cooking conditions did not pass 92 °C and were not prolonged for more than four minutes. These conditions were selected to best simulate domestic conditions using an electric device set at medium cooking heat. Most studies on chicken meat burgers have tested grilled or oven-cooked patties. Other references on chicken meat evaluated entire boiled or roasted chicken. This is a fundamental step in explaining, for example, the absence of sulphur containing volatiles. In line with findings of other researchers [53], these compounds come from the interaction among Maillard reaction compounds and lipid oxidation products. Thus, quick cooking processes, medium/low heating, or their combination seem not to favour this interaction. These settings did not allow the development of traceable Maillard reaction products (desired and undesired). T_72_ (CTRL) samples showed an increase in concentration of hexanal, the emergence of heptanal, some alcohols such as 2-Nonen-1-ol, (E)-, ketones as 2,3-Octanedione and 7,9-Di-tert-butyl-1-oxaspiro (4,5) deca-6,9-diene-2,8-dione, and just one Sulphur containing compound: N-Methyl-taurine. These are all markers of lipid oxidation and muscle damage. The addition of by-products provoked a mitigation of some WOF species, but also gave to patties specific odorants not conducible to meat oxidation and potentially undesirable.

CSS addition reduced the occurrence of aldehydes such as hexanal that reduced from 55.1% (CTRL T_0_) to 11.7% (CSS T_0_ 1.5%) to 0 (CSS T_0_ 3%). At T_72_ CTRL contained 72% and reduced to 0 in both concentrations. Heptanal was found only in CTRL T_72_; octanal too, was just found in CTRL samples and not found in samples 1.5 and 3% at both times. Some alcohols such as 2-Nonen-1-ol, (E)- were limited in T_72_ samples, but were present in T_0_ CSS 3% with other alcohols, probably from the degradation of lignocellulosic precursors. This same pathway is followed by hydrocarbons which are totally absent in CTRL samples while being present in added patties [54]. According to data here shown, Nitrogen containing compounds present in CSS formulations probably came from the degradation of CSS proteins and from the Maillard complex of reactions which takes place during the roasting process. As for CSS, the significant role of phenols’ interaction with Maillard reaction products to produce specific compounds can be assumed. Unfortunately, CSS developed p-xylene and o-xylene, involved in the rise of WOF and referred to as “cardboard-like” [55,56]. No references are available to compare results obtained, especially for CSS properties.

Chromatograms, in all cases, showed a great reduction of WOF or general active odorants. As a demonstration of this, CTRL T_0_ and T_72_ images had a resolution with an order of magnitude of 10^10,^ while CSS had values of 10^8^. From pictures, the peak of hexanal that eluted at around 6.48 min is always visible and it is clear how much it decreases in respect of additions of by-products. In all samples at around minutes 13.44 and 16.49, long chain aldehydes were eluted (i.e., octanal, decenal). At around minutes 19.35–37 2-Nonen-1-ol, (E)- was eluted in almost all samples. After minute 19.40, the main compounds traced were siloxanes and low matched compounds.

Of course, a better characterization of the aromatic profile of these molecules is fundamental to understand if and how volatiles from these substrates can have a negative impact on the final flavour of meat products.

#### 3.2.3. Sensory Analysis

Panellists involved in this analysis were asked to try samples T_0_ and T_72_ of all formulations on separate days without knowing what they were eating, with the objective of evaluating the presence and the intensity of WOF markers and possible perceptions of extraneous flavours in cooked burgers after refrigerated storage. Descriptors were carefully explained, especially those difficult to associate with food, such as “cardboard-like” or “paint”.

In Table 5 are reported all the average values for burger samples tasted immediately after cooking (T_0_) and after 72 h of refrigerated storage (T_72_).

T_0_ samples of all formulations did not show significative differences, and panellists were not able to trace significant variability from control. Statistical analysis did not show significance between samples even if, looking at the average values, some considerations can be made. The score reported for the bitter descriptor rose to 2.25 for the 3% CSS formulation, while CTRL was 1.25 and CSS 1.5% was 1.87. CSS addition did not influence the average score for all descriptors, and, in most cases, they were the same as CTRL or very near to it. CSS seemed to influence the perception of cooked meat odour rising from 1.75 in CTRL, to 2.87 in 1.5% addition, to 2.75 in 3% addition. This is one of the few situations in which 1.5% received higher scores than 3%. Astringency also registered an increase from CTRL to the 1.5% CSS addition of 3%: from 1.62 to 1.75 to 2.37, respectively.

Generally, all the descriptors for all formulations received increased scores, but some significant differences were traced via the statistical analysis or can be noted from the direct comparison among the average scores. Only two descriptors for CSS formulations were significant (*p* < 0.05): cooked meat odour and bitter. Cooked meat odour was mitigated, mainly by CSS addition. For this descriptor, the score decreased from 4 (CTRL) to 2.75 (1.5%) and 2.62 (3%). This limitation can be seen neither as negative nor positive. If the cooked meat odour can be directly linked with positive sensations, we do not know the considerations of each panellist regarding cooked meat. Nevertheless, from the explanation of each descriptor and the training, these lower scores do not directly show a positive thing. For better discrimination, a comparison with the roasted descriptor can be made; although not significant, it received lower scores than cooked meat.

The presence of this descriptor was not casual, because it helped in separating what flavours, odours, and aromas can come from a fresh grilled or oven/pan-cooked burger, instead of an already cooked and reheated burger. Panelists were not aware of the cooking process. The ‘roasted’ adjective generally includes those positive flavours coming from the Maillard complex of the reaction, while cooked meat is mainly linked with sensations of staling. In these terms, the mitigation obtained from by-products can be positive, while increasing the stability of the product. Bitterness perceived in CSS formulated patties can be a result of the reheating of meat. The data show an increase from CTRL at 1.37, to 1.62 (CSS 1.5%), and to 2.35 (CSS 3%). Heat can make the condensation or splitting of phenolic species easier. During the roasting process, chlorogenic acids degrade to active taste lactones which give desirable sourness and bitterness [57]. When exposed to further heating, these species undergo greater degradation which leads to the splitting of quinic acid which, in successive steps will give metallic, lingering bitter phenyl-indanes which are undesirable for coffee taste. Caffeine and, in general, methylxanthines-alkaloids give a bitter and astringent note. Probably, the double exposure to heating, even if at lower temperatures, can determine higher bitterness.

Refrigerated storage can also favour the condensation of flavonoids to tannins or bigger phenolic species, but no sources are available at this time. A general positive comment is that none of the descriptors directly linked with the development of WOF (paint, cardboard-like, vegetable oil-like and sulphur/rubber) was increased. Even if not significative, average scores of the CSS added sample were lowered in respect of CTRL. Control had 2, while CSS fell to 1.75 for 1.5% addition, and to 1.5 for 3% addition. Acidity and even metallic sensations were not increased. To better investigate the significance of cooked meat flavour and bitterness, different factors were considered. Time, Formulation, and Time x Formulation were selected as factors. For cooked meat flavour, the time factor, i.e., the effect of time, was significant for *p* < 0.01 while time x formulation factor had a *p* < 0.05 (Table 6). Formulation alone did not influence the results. Conversely, bitterness was influenced only by the formulation with a *p* < 0.05. Neither time nor time x formulation factors made an effect. Overall, T_0_ samples did not show any significant difference for the cooked meat odour descriptor. After 72 h of refrigerated storage, the time effect greatly influences (*p* < 0.01) this characteristic.

## 4. Conclusions

The present study provides data on technological performance, nutritional aspects and effects on stability of newly formulated meat products.

Coffee silver skin could be considered as a food ingredient that can solve a complex problem in limiting the decay of meat foods (especially of poultry origin) and lowering the food waste caused by coffee production. CSS can become a cheap but valuable integrator of fibres and bioactive molecules; moreover, it is a great source of minerals such as calcium, potassium, and others.

Data obtained are the starting point of a deeper study to comprehend what are the best conditions to use this by-product and how to develop “tailor-made” formulations enjoyable to consumers. Burgers were among the easiest preparations, allowing a direct comparison with reality.

CSS has shown potential for being implemented in meat formulations to limit losses connected with the cooking process.

Considering the principal aim of this study (to understand the role of CSS on WOF occurrence), encouraging results are provided. The sensory analysis conducted gave confirmation that the spreading of WOF, or in general of oxidation markers, was arrested, even if bitterness and astringency can emerge with time. The inclusion of CSS among new ingredients for food production is hoped for, even if further analysis is needed with further consultation procedures regarding current novel food statuses.

## Figures and Tables

**Table 1:** Proximate composition (mean ± standard deviation, SD) of raw burger samples (before cooking): control (CTRL), with addition of +1.5% of coffee silver skin (CSS 1.5%) and +3.0% of coffee silver skin (CSS 3.0%).

Sample	Moisture (%)	Proteins (%)	Lipids (%)	Ashes (%)
CTRL	74.56 ± 0.05	19.72 ± 0.05	2.48 ± 0.01	1.97 ^a^ ± 0.01
CSS 1.5%	74.14 ± 0.03	20.01 ± 0.23	2.74 ± 0.02	2.11 ^b^ ± 0.01
CSS 3%	73.39 ± 0.05	19.62 ± 0.05	2.69 ± 0.01	2.69 ^c^ ± 0.01
sign.	n.s.	n.s.	n.s.	**

Legend: data followed by different superscript letters, in the same column, are significantly different (least significant difference (LSD) test, *p* < 0.05); asterisks indicate significance at ** *p* < 0.01, n.s. not significant.

**Table 2:** Results (mean ± SD) of pH, water activity (a_w_) values and cooking yield (%) in burger samples (CTRL, control; CSS 1.5% and CSS 3.0%, with +1.5% and +3.0% of coffee silver skin as ingredient, respectively).

	pH _Uncooked_	pH _Cooked_	a_w Cooked_	Cooking Yield %
CTRL	5.82 ± 0.03	6.10 ± 0.03	0.985 ± 0.0013	76.15 ^a^ ± 0.58
CSS 1.5%	5.75 ± 0.01	6.06 ± 0.01	0.986 ± 0.0016	89.5 ^b^ ± 0.36
CSS 3%	5.75 ± 0.01	6.02 ± 0.01	0.986 ± 0.0007	88.95 ^b^ ± 0.52
sign.	n.s.	n.s.	n.s.	**

Legend: data followed by different superscript letters, in the same column, are significantly different (LSD test, *p* < 0.05); asterisks indicate significance at ** *p* < 0.01; n.s. not significant.

**Table 3:** Colour values (mean ± SD) of L* (lightness), a* (redness), b* (yellowness), and C* (chroma) for uncooked burgers and cooked burgers, in control (CTRL) and in samples with +1.5% (CSS 1.5%) and +3.0% (CSS 3.0%) of coffee silver skin as ingredient.

Uncooked Samples	L*	a*	b*	C*
CTRL	45.91 ± 0.18 ^a^	0.28 ± 0.11 ^b^	10.95 ± 0.53	10.96 ± 0.53 ^b^
CSS 1.5%	32.83 ± 1.33 ^b^	3.81 ± 1.21 ^a^	13.74 ± 1.58	15.42 ± 1.82 ^a^
CSS 3%	29.40 ± 0.71 ^c^	4.96 ± 0.83 ^a^	14.60 ± 0.31	14.28 ± 0.52 ^a^
sign.	**	**	n.s.	*
**Cooked Samples**				
CTRL	56.12 ± 0.76 ^a^	1.18 ± 0.77	16.75 ± 0.49 ^a^	16.80 ± 1.08 ^a^
CSS 1.5%	47.35 ± 0.44 ^b^	2.25 ± 0.60	10.94 ± 0.36 ^b^	11.17 ± 0.42 ^b^
CSS 3%	43.54 ± 0.27 ^b^	2.83 ± 0.60	12.05 ± 0.40 ^b^	12.38 ± 1.22 ^b^
sign.	*	n.s.	**	**

Legend: data followed by different superscript letters, in the same column, are significantly different (LSD test, *p* < 0.05); asterisks indicate significance at * *p* <0.05; ** *p* < 0.01; n.s. not significant.

**Table 4:** Qualitative profile of main volatile compounds (area %) in cooked samples (control, addition of coffee silver skin 1.5 and 3%), immediately after cooking process (T_0_) and after 72 h of storage at +4 °C (T_72_).

VOCCs from Burgers’ Samples		Burgers T_0_			Burgers T_72_		
	CTRL T_0_	CSS 1.5% T_0_	CSS 3% T_0_	CTRL T_72_	CSS 1.5% T_72_	CSS 3% T_72_	Sign.
Aldehydes							
2-Decenal, (Z)-	nd	nd	1.25	nd	nd	nd	*
2-Nonenal, (E)-	nd	nd	nd	nd	2.82	nd	*
10-Undecenal	nd	nd	4.06	nd	nd	nd	*
Benzaldehyde	nd	6.27 ^c^	4.46 ^b^	nd	3.62 ^a^	7.42 ^d^	*
Benzaldehyde, 2,5-bis[(trimethylsilyl)oxy]-	0.86 ^a^	nd	nd	nd	0.72 ^a^	1.73 ^b^	*
Butanal, 2-methyl-	nd	nd	5.48 ^a^	nd	nd	46.86 ^b^	**
Butanal-3 methyl	nd	nd	5.67 ^a^	nd	4.11 ^a^	7.00 ^b^	*
Heptanal			1.77 ^a^	4.46 ^b^	2.29 ^a^	nd	*
Hexanal	55.1 ^b^	11.7 ^a^	nd	72 ^c^	nd	nd	***
Octanal	41.2 ^b^	nd	nd	7.94 ^a^	nd	nd	*
Pentanal	2.09 ^a^	nd	nd	nd	nd	nd	*
Propanal, 2-methyl-	nd	nd	3.34 ^B^	nd	nd	1.98 ^a^	*
Alcohols							
1,5-Pentanediol, 3-methyl-	nd	nd	8.96 ^b^	nd	nd	3.90 ^a^	*
1-Methylcyclopropanemethanol	nd	nd	nd	nd	nd	3.71 ^a^	*
2-Hexen-1-ol, (Z)-	nd	nd	nd	nd	10.52	nd	*
3-Decyn-2-ol	nd	nd	nd	nd	5.76	nd	*
2-Nonen-1-ol, (E)-	0.58 ^a^	10.76 ^b^	8.38 ^a^	8.80 ^a^	nd	nd	*
2-Octen-1-ol, (E)-	nd	nd	2.03	nd	nd	nd	*
Ketones							
2,3-Octanedione	nd	nd	nd	5.14	nd	nd	*
7,9-Di-tert-butyl-1-oxaspiro (4,5) deca-6,9-diene-2,8-dione	nd	nd	3.78 ^b^	0.20 ^a^	nd	nd	*
Nitrogen-containing compounds							
2-(Aziridinylethyl)amine	nd	16.09 ^c^	7.43 ^b^	nd	15.24 ^c^	4.59 ^a^	*
2,6,6-Trimethyl-bicyclo [3.1.1]hept-3-ylamine	nd	nd	nd	nd	4.42	nd	*
Benzeneethanamine, 2,5-difluoro-β,3,4-trihydroxy-N-methyl-	nd	nd	4.37	nd	nd	nd	*
Oxime-, methoxy-phenyl-_	nd	nd	nd	nd	nd	1.48	*
Propanamide, 2-hydroxy-	nd	nd	8.86 ^a^	nd	20.18 ^b^	nd	*
Topotecan	nd	nd	nd	nd	8.81	nd	*
Hydrocarbons							
2-Trifluoroacetoxydodecane	nd	3.98	nd	nd	nd	nd	*
3-Trifluoroacetoxydodecane	nd	4.35	nd	nd	nd	nd	*
Butane, 2-nitro-	nd	3.74	nd	nd	nd	nd	*
Cyclohexene, 1-methyl-4-(1-methylethenyl)-, (S)-	nd	nd	nd	nd	nd	3.43	*
Ergosta-5,22-dien-3-ol, acetate, (3β,22E)-	nd	nd	nd	nd	10.26	nd	*
Ethylbenzene	nd	nd	1.56 ^a^	nd	nd	4.17 ^b^	*
Hydroperoxide, heptyl	nd	3.87	nd	nd	nd	nd	*
Propane	nd	nd	nd	nd	nd	4.05	*
p-Xylene	nd	2.57 ^a^	nd	nd	2.48 ^a^	3.00 ^b^	*
Esters							
1,2-Benzenedicarboxylic acid, butyl octyl ester	nd	nd	nd	0.04	nd	nd	*
Other compounds							
2-Formylhistamine	nd	nd	nd	nd	5.55	nd	*
Butylated Hydroxytoluene	nd	nd	nd	0.20	nd	nd	*
Nitrous oxide	nd	nd	3.91	nd	nd	nd	*
Pregnan-18-oic acid,3,11,21-trihydroxy-20-oxo-, γ-lactone, (3β,5α,11β)-	nd	nd	1.24	nd	nd	nd	*

Legend: nd, not detectable; data followed by different superscript letters, in the same row, are significantly different (LSD test, *p* < 0.05); asterisks indicate significance at * *p* < 0.05; ** *p* < 0.01; *** *p* < 0.001.

**Table 5:** Average scores for cooked CSS containing samples of panelists for each descriptor in all formulations; immediately after cooking process (T_0_) and after 72 h of refrigerated (+4 °C) storage (T_72_).

Average Scores	CTRL		CSS 1.5%		CSS 3%	
	0 h	72 h	0 h	72 h	0 h	72 h
Descriptors						
cooked meat odour	1.8 ± 0.9 ^b^	4 ± 1.4 ^a^	2.9 ± 1.1 ^a^	2.7 ± 1.0 ^ab^	2.8 ± 1.1 ^a^	2.6 ± 0.7 ^ab^
cardboard	1.9 ± 0.8	1.7 ± 0.9	1.5 ± 0.7	2.0 ± 0.5	1.6 ± 0.9	2.0 ± 0.1
Sulphur/rubber	1.6 ± 1.0	2.0 ± 1.0	1.3 ± 0.4	1.5 ± 1.0	1.3 ± 0.4	1.5 ± 0.7
roasted	2.1 ± 1.1	1.7 ± 0.7	2.6 ± 0.7	1.8 ± 0.8	2.8 ± 1.3	2.5 ± 0.9
painty	1.0 ± 0.0	1.3 ± 0.7	1.0 ± 0.0	1.1 ± 0.3	1.5 ± 1.0	1.2 ± 0.4
rancid	1.6 ± 1.0	1.5 ± 0.7 ^a^	1.0 ± 0.0	1.1 ± 0.3	1.2 ± 0.3	1.3 ± 0.7
vegetable oil-like	1.6 ± 0.7	1.6 ± 0.7	1.6 ± 0.9	1.3 ± 0.5	1.6 ± 0.7	1.6 ± 0.7
sour	1.8 ± 0.7	1.2 ± 0.4 ^a^	1.9 ± 0.8 ^a^	1.6 ± 0.7	1.6 ± 0.9	1.6 ± 1.0
bitter	1.3 ± 0.5 ^b^	1.4 ± 0.2 ^b^	1.9 ± 1.0 ^ab^	1.6 ± 0.7 ^ab^	2.6 ± 0.3 ^a^	2.3 ± 0.9 ^a^
metallic	1.3 ± 0.5	1.1 ± 0.3	1.4 ± 0.7	2.0 ± 1.2 ^a^	1.3 ± 0.4	1.8 ± 1.1
astringent	1.6 ± 0.7	1.8 ± 0.7	1.8 ± 0.9	1.7 ± 0.9	2.4 ± 1.0	2.6 ± 0.8

Legend: data followed by different superscript letters, in the same row, are significantly different (LSD test, *p* < 0.05).

**Table 6:** Anova matrix results for significant descriptors (cooked meat odour and bitterness).

	Cooked Meat Odour		Bitterness	
Factor	F	sign.	F	sign.
Formulation	1.8019	n.s.	3.7026	*
Time	14.6049	**	0.7326	n.s.
Formulation × Time	3.4424	*	0.4273	n.s.

Legend: asterisks indicate significance at * *p* <0.05; ** *p* < 0.01; n.s. not significant.

## Supplementary Materials

The following are available online at https://www.mdpi.com/article/10.3390/foods10081833/s1, Figure S1: Chromatogram of volatile compounds (VOCs) in chicken burgers immediately after cooking (a, CTRL T_0_) and after 72 h of refrigerated storage (b, CTRL T_72_); Figure S2: Chromatogram of volatile compounds (VOCs) in chicken burgers formulated with CSS 1.5%, immediately after cooking (a) and after 72 h of refrigerated storage (b); Figure S3: Chromatogram of volatile compounds (VOCs) in chicken burgers formulated with CSS 3%, immediately after cooking (a) and after 72 h of refrigerated storage (b).
