# Supplementary material for: The Role of Coffee Silver Skin against Oxidative Phenomena in Newly Formulated Chicken Meat Burgers after Cooking
Source: Foods. 2021 Aug 8;10(8):1833. doi: 10.3390/foods10081833 (PMC8394139; doi:10.3390/foods10081833)
Supplement: Supplementary file 1 [file foods-10-01833-s001.zip › foods-1303890-supplementary.pdf]

Supplementary Materials

(**a**)

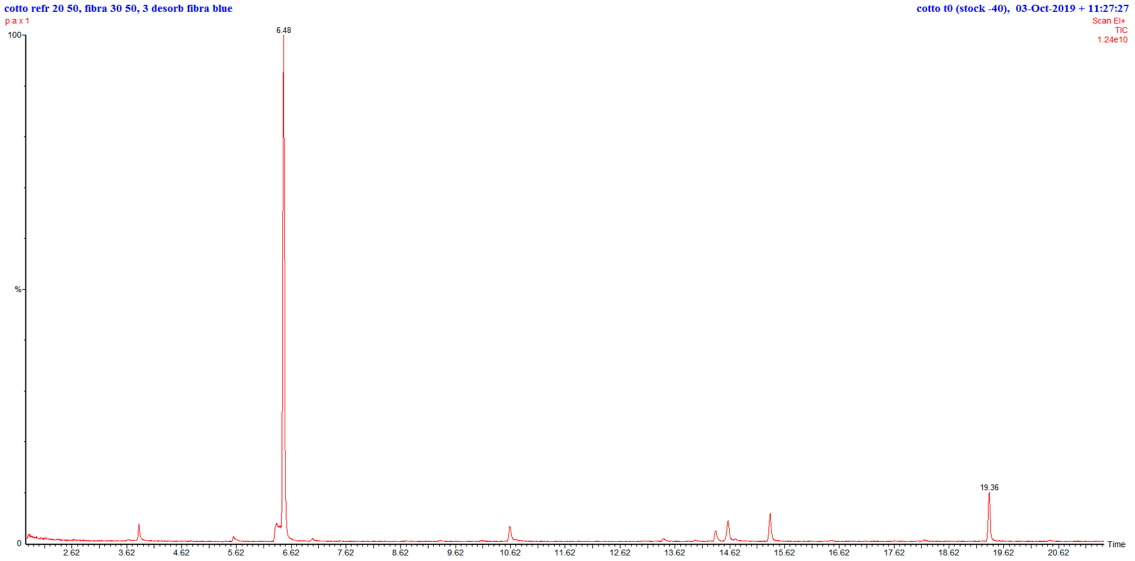

(**b**)

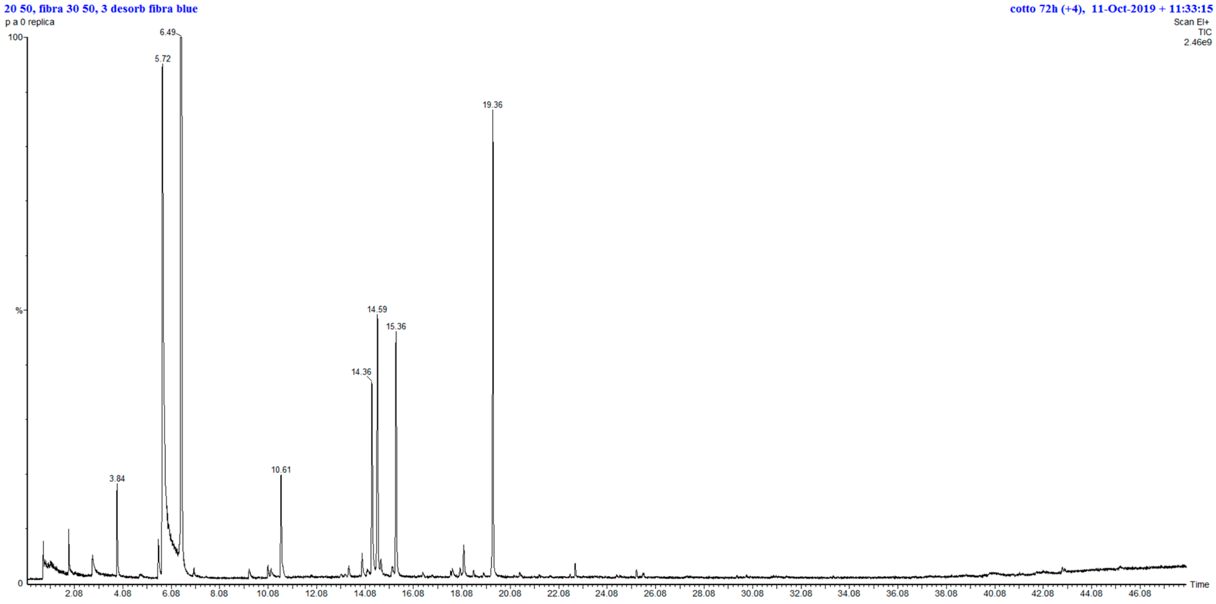

Figure S1. Chromatogram of volatiles compounds (VOCs) in chicken burgers immediately after cooking (**a**, CTRL T_0) and after 72 hours of refrigerated storage (**b**, CTRL T_{72}).

(**a**)

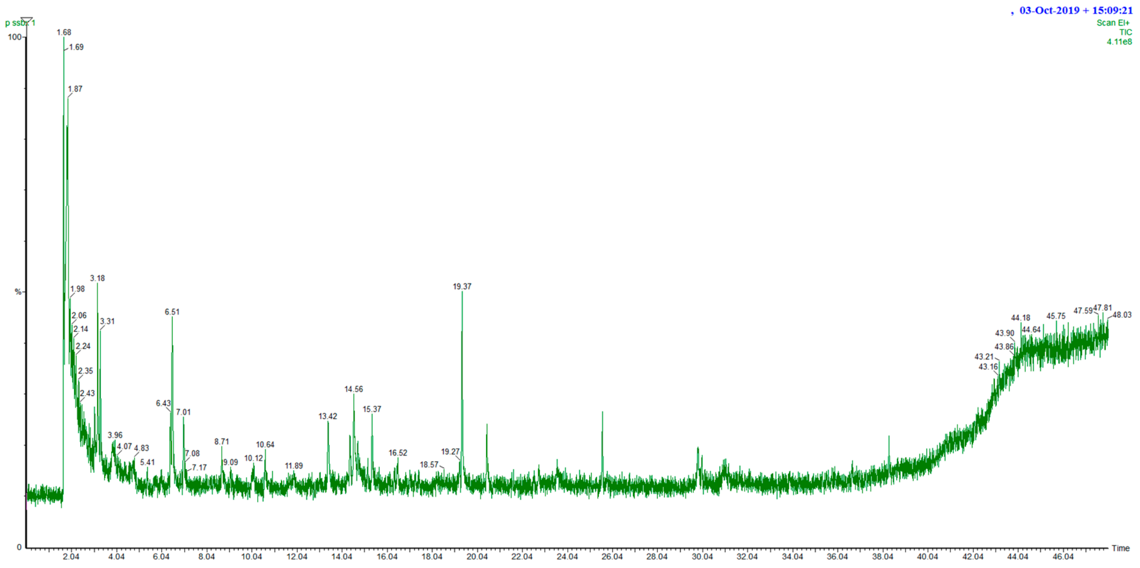

(**b**)

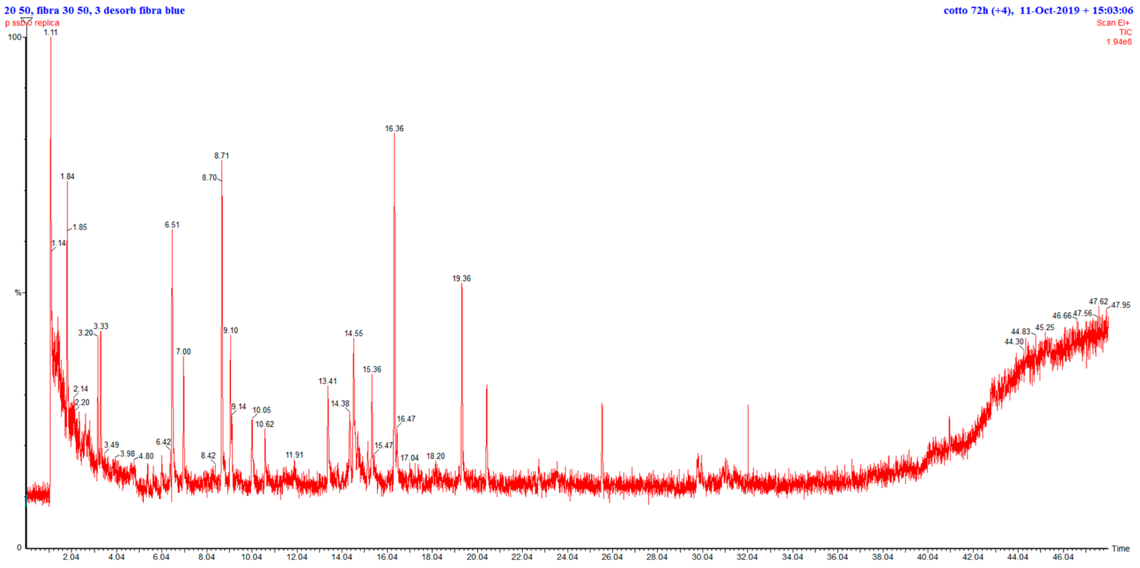

Figure S2. Chromatogram of volatiles compounds (VOCs) in chicken burgers formulated with CSS 1.5%, immediately after cooking (**a**) and after 72 hours of refrigerated storage (**b**).

(**a**)

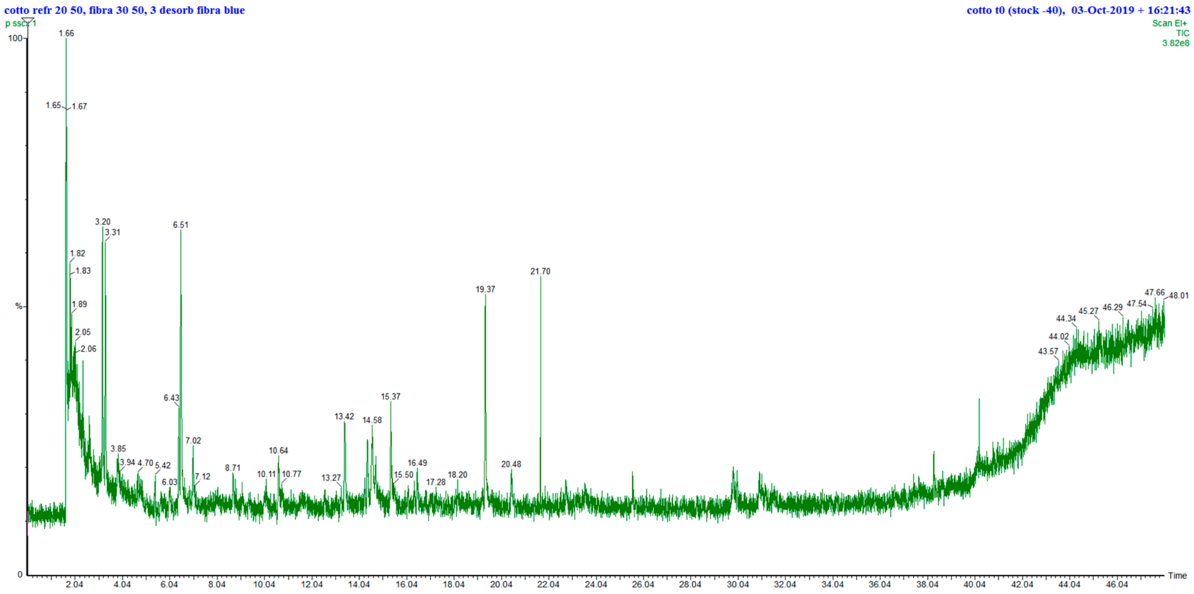

(**b**)

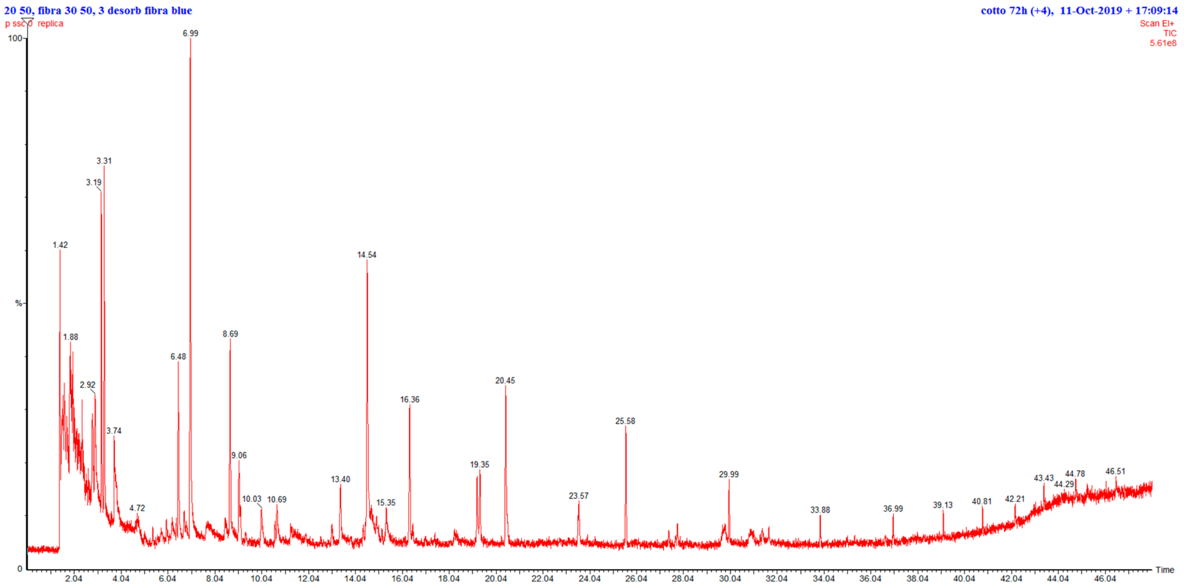

Figure S3. Chromatogram of volatiles compounds (VOCs) in chicken burgers formulated with CSS 3%, immediately after cooking (**a**) and after 72 hours of refrigerated storage (**b**).
